# Novel protective circulating miRNA are associated with preserved vitamin D levels in patients with mild COVID-19 presentation at hospital admission not progressing into severe disease

**DOI:** 10.1007/s12020-024-03900-6

**Published:** 2024-06-10

**Authors:** Luigi di Filippo, Umberto Terenzi, Giovanni Di Ienno, Silvia Trasciatti, Silvano Bonaretti, Andrea Giustina

**Affiliations:** 1 Institute of Endocrine and Metabolic Sciences, San Raffaele Vita Salute University and IRCCS San Raffaele Hospital, Milan, Italy; 2 Galileo Research Srl, Pisa, Italy

**Keywords:** Vitamin D, Vitamin D deficiency, COVID-19, miRNA, SARS-CoV-2

## Abstract

**Purpose:**

Low vitamin D levels were reported to negatively influence the outcomes of acute COVID-19, as well as other biochemical markers were linked to COVID-19, including microRNAs (miRNAs). This study aimed to prospectively evaluate miRNAs and vitamin D relationship in predicting COVID-19 outcomes.

**Methods:**

COVID-19 patients were part of a previously reported cohort and enrolled in a matched-ratio based on the presence/or not of severe disease at hospital admission. 25(OH) vitamin D levels and miRNAs expression were evaluated.

**Results:**

Patients affected by non-severe COVID-19 were characterized by a higher expression of miRNAs hsa-miR-3115 and hsa-miR-7151-3p, as compared to those affected by severe disease. In non-severe patients, these miRNAs were more frequently expressed in those who subsequently did not develop worsening outcomes. In addition, patients with miRNA-7151 expression and without worsening disease were characterized by higher 25(OH) vitamin D levels and lower prevalence of vitamin D deficiency.

**Conclusions:**

The expression of two novel miRNAs was reported for the first-time to be associated with a less severe COVID-19 form and to prospectively predict the occurrence of disease outcome. Furthermore, the association observed between vitamin D deficiency and lack of miRNA-7151 expression in COVID-19 patients with worse outcomes may support the hypothesis that the co-existence of these two conditions may have a strong negative prognostic role.

## Introduction

Coronavirus disease 2019 (COVID-19) manifestations are known to range from asymptomatic forms to acute respiratory-distress syndromes with high mortality risk. A proper identification of severe forms and an early prediction of high-risk patients is still required. Therefore, several clinical and biochemical features have been identified and proposed as useful tools in predicting disease severity and future outcomes. In the context of the endocrine phenotype of the disease [1, 2], the negative role of vitamin D deficiency was hypothesized early due to its the well-known influence on immune response and immunocompetence [3–5]. Lower vitamin D levels were reported consistently to be associated with severe COVID-19, and also demonstrated to prospectively predict the occurrence of worse outcomes independently from the severity of disease at presentation [6]. In addition, among the most promising biochemical markers in predicting COVID-19 severity, also those involved in regulation of gene expression such as microRNAs (miRNAs) were reported consistently [7, 8]. MiRNAs are small noncoding RNAs that play a key-role in various biological and pathological processes that in the last decades have been progressively proposed as diagnostic and predicting biomarkers for several diseases, including infectious ones. Their physiological role is to silence gene expression through messenger-RNA inhibition. Several studies and meta-analyses have addressed miRNAs’ role in COVID-19, highlighting consistent associations between their expression and susceptibility to worse outcomes [7, 8]. Our aim was to report the results of an ancillary study of a previously published observational trial [6] on miRNAs expression in COVID-19 patients carrying out also, for the first-time, a prospective evaluation of miRNAs and vitamin D relationship in predicting COVID-19 outcomes.

## Patients and methods

### Study design

We performed a prospective ancillary study with post-hoc analyses on a cohort of patients evaluated at San Raffaele University Hospital, Milan, Italy, and previously described (hospital ethics committee protocol: ABIO/NC/03 no. 367/2020) [6]. Study design was detailed in our previous report. Briefly, adult patients (age ≥18 years) admitted for COVID-19 from March to June 2021 were evaluated for the enrollment. At admission in Emergency-Department (ED), patients were consecutively enrolled in a matched for age, sex and comorbidities 1:1 ratio based on the presence or not of respiratory-distress (complete list of criteria detailed in [6]) defining those with severe and non-severe disease. Worsening disease was defined by the subsequent occurrence of severe clinical course in patients initially characterized as non-severe in ED [6].

### Data collection

Data collected included demographics, comorbidities, inflammatory parameters, and clinical outcomes (complete list in [6]). 25(OH) vitamin D was measured, at study enrollment in ED, by Roche Cobas 8000WKC/MET/036 using electrochemiluminescence-immunoassays (ECLIA) (ng/mL) (coefficient of variation 5%). Vitamin D deficiency was defined as 25(OH) vitamin D levels below 20 ng/mL. 25(OH) vitamin D data of the patients evaluated in this ancillary study were already included in the core trial previously published [6]. The expression levels of circulating miRNAs were estimated using Next Generation Sequencing system (Illumina) on patients’ plasma samples collected at admission. RNA was isolated using miRNeasy Serum/Plasma Kit (Qiagen) and the RNA Library was prepared using NEXTFlex Small RNA-Seq Kit v3 (Bioo Scientific).

**Fig. 1:**
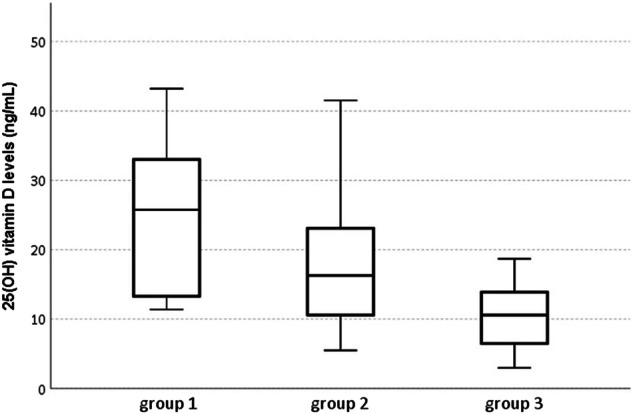
25(OH) vitamin D levels across patients with miRNA-7151 expression and without worsening disease (group 1) (n.6), patients without both miRNA-7151 expression and worsening disease (group 2) (n.21), and patients without miRNA-7151 expression but with occurrence of worsening disease (group 3) (n.9)

On the basis of occurrence or not of worsening disease and concomitant presence or absence of the specific miRNA expression, the patient’s cohort was also retrospectively subdivided into three groups compared in Fig. 1. Group 1: without occurrence of worsening disease and with miRNA expression; group 2: without occurrence of worsening disease and without miRNA expression; group 3: with occurrence of worsening disease and without miRNA expression. Only one patient with occurrence of worsening disease and with miRNA-7151 expression was observed (data not included in the analysis for statistical reasons).

### Statistical analysis

Descriptive statistics were obtained for all study-variables. Categorical variables were summarized as counts and percentages. Kolmogorov–Smirnov normality-test was performed (*p* < 0.05) and continuous variables were expressed as medians and interquartile range [IQR] [25th–75th percentile]. Fisher exact test or χ2 test and the Wilcoxon signed-rank test or the Kruskal–Wallis test were used to determine the statistical significance of differences in proportions and medians, respectively. All statistical tests were two-sided. *P* value of <0.05 was considered statistically significant. Analysis was conducted using IBM SPSS Statistics (Statistics for Windows, 23.0, IBM Corp.).

## Results

As previously described, a total of 73 patients were prospectively enrolled including 36 (49.3%) and 37 (50.7%) with and without severe disease at hospital admission, respectively [6]. Briefly, median age of patients was 68 [54–73] years, 43 (58.9%) were male, and history of hypertension was the most frequent comorbidity (57.5%). Median 25(OH) vitamin D level was 13.8 [8.8–20.1] ng/mL and vitamin D deficiency was found in 55 patients (75%) [6].

The evaluation of levels of circulating miRNAs revealed that patients affected by non-severe COVID-19 were characterized by more frequent expression of miRNAs hsa-miR-3115 and hsa-miR-7151-3p, as compared to those affected by severe disease.

As previously reported, in the non-severe group (n.37), a worsening disease subsequently occurred in 10 patients (27%) [6]. MiRNA-3115 was found in a total of 9 patients, of whom no one developed a worsening disease (0/10 vs 9/27, *p* = 0.079). MiRNA-7151 was found in a total of 7 patients, of whom only one showed a worsening disease (1/10 vs 6/27, *p* = 0.65). No differences were observed between those with and without the expression of miRNA-3115 or miRNA-7151 regarding demographics and comorbidities (Table 1; Table 2). However, subjects in whom those miRNAs were found were also characterized by statistically significant lower levels of inflammatory biomarkers (Table 1; Table 2).

**Table 1:** Demographic characteristics, concomitant comorbidities and disease outcomes in COVID-19 patients with and without miRNA-3115 expression

	miRNA-3115 + (n.9)	miRNA-3115 – (n.28)	p value
Age, years	61 (52–73)	64 (52–73)	0.99
Male gender, *n* (%)	6 (66%)	17 (61%)	0.99
BMI, kg/m^2^	27 (26–31)	26 (25–30)	0.22
Obesity, *n* (%)	2 (22%)	5 (18%)	0.99
Hypertension, *n* (%)	6 (66%)	16 (57%)	0.71
Cardiovascular disease, *n* (%)	0 (0%)	3 (11%)	0.56
Diabetes mellitus, *n* (%)	3 (33%)	3 (11%)	0.14
History of neoplasia, *n* (%)	1 (11%)	1 (3%)	0.43
25(OH) vitamin D levels, ng/mL	14.1 (10.1–21.8)	13.2 (8.4–35)	0.96
Vitamin D deficiency, *n* (%)	6 (66%)	20 (71%)	0.99
LDH, U/L	258 (206–327)	327 (251–350)	**0.021**
CRP, mg/L	41 (13–64)	57 (17–79)	0.29
Ferritin, ng/mL	474 (118–860)	723 (427–994)	**0.012**
IL6, pg/mL	0.19 (0.09–1)	0.97 (0.53–3.1)	**0.015**
Worsening disease, *n* (%)	0 (0%)	10 (36%)	0.079

P values reported in bold are statistically significant

*n* number, *BMI* body mass index, *LDH* lactate dehydrogenase, *CRP* c-reactive protein, *IL6* interleukin 6

**Table 2:** Demographic characteristics, concomitant comorbidities and disease outcomes in COVID-19 patients with and without miRNA-7151 expression

	miRNA-7151 + (n.7)	miRNA-7151 – (n.30)	p value
Age, years	60 (53–74)	65 (51–73)	0.89
Male gender, *n* (%)	4 (57%)	19 (63%)	0.99
BMI, kg/m^2^	27 (26–29)	26 (25–30)	0.61
Obesity, *n* (%)	1 (14%)	6 (20%)	0.99
Hypertension, *n* (%)	4 (57%)	18 (60%)	0.99
Cardiovascular disease, *n* (%)	1 (14%)	2 (7%)	0.48
Diabetes mellitus, *n* (%)	0 (0%)	6 (20%)	0.57
History of neoplasia, *n* (%)	0 (0%)	2 (7%)	0.99
25(OH) vitamin D levels, ng/mL	20.6 (11.4–33)	13.5 (9.5–20.3)	0.11
Vitamin D deficiency, *n* (%)	3 (43%)	23 (76%)	0.16
LDH, U/L	278 (227–307)	306 (271–345)	0.41
CRP, mg/L	31 (11–67)	58 (20–75)	**0.031**
Ferritin, ng/mL	565 (215–885)	658 (385–901)	0.35
IL6, pg/mL	0.34 (0.14–3)	1.2 (0.8–4.1)	**0.041**
Worsening disease, *n* (%)	1 (14%)	9 (30%)	0.65

P values reported in bold are statistically significant

*n* number, *BMI* body mass index, *LDH* lactate dehydrogenase, *CRP* c-reactive protein, *IL6* interleukin 6

In non-severe patients, we had previously reported that, in multivariate analyses, lower 25(OH) vitamin D levels resulted as the only variable independently associated with the occurrence of worsening disease [6]. Regarding vitamin D and miRNAs relationship, no differences in 25(OH) vitamin D levels and vitamin D deficiency prevalence were observed between those with miRNA-3115 expression and without worsening disease (n.9) vs those either without both miRNA-3115 expression and worsening disease (n.18) or without miRNA-3115 expression but with occurrence of worsening disease (n.10) (Table 1). On the other hand, 25(OH) vitamin D levels were significantly higher in those with miRNA-7151 expression and without worsening disease (n.6) as compared to those without both miRNA-7151 expression and worsening disease (n.21) and to those without miRNA-7151 expression but with occurrence of worsening disease (n.9) (25.7 [12.8–35.5] vs 16.3 [10.3–25.9] vs 10.6 [6.1–14.2] ng/mL, respectively, *p* = 0.022) (Fig. 1). In addition, vitamin D deficiency prevalence was lower in those with miRNA-7151 expression and without worsening disease as compared to the other two groups (33% vs 66% in group 2 and vs 100% in group 3, *p* = 0.021).

## Discussion

In our previously reported cohort, miRNA-3115 and miRNA-7151 were expressed in non-severe group exclusively. In addition, patients who expressed miRNA-3115 and miRNA-7151 were characterized by a lower inflammatory response, and these miRNAs were present more prevalently in those who did not develop a subsequent worsening disease as compared to those who worsened.

To date, no data are available on miRNA-3115 and miRNA-7151 effects in COVID-19 and our data seem to potentially support, for the first-time, a possible protective role of these miRNAs in COVID-19 patients. The pathophysiological roles of miRNA-3115 and miRNA-7151 are currently poorly understood. Interestingly, using a widely accepted database for prediction of miRNAs functional targets, we have evaluated candidate transcripts with very high scores ≥80 (range 0–100) observing that miRNA-3115 is potentially related to a negative modulation of G-protein-coupled receptor 17 (GPR17) and miRNA-7151 to an inhibitory effect on cathepsins B/L (CTSB/L) and Kringle Containing Transmembrane Protein 1 (KREMEN1) [9]. Interestingly, GPR17 is a seven-transmembrane domains receptor with a tissue broad distribution playing different roles in cells remodeling and repairing [10]. Recently, the inhibition of GPR17 in mouse models using Cangrelor, an antagonist anti-platelet drug, was associated with a significant decrease in the inflammatory response injury and pulmonary fibrosis during sepsis [11]. Also, CTSB/L is an endosomal protease involved in cleaving and activating SARS-CoV-2 spike-protein, a required step for viral entry in host-cells [12, 13]. In vitro studies have demonstrated that blocking CTSB/L effectively reduces and prevents viral entry [12, 13]. Finally, KREMEN1 is a transmembrane protein expressed in various tissues and, recently, was also demonstrated to act as an ACE2-alternative viral entry receptor for SARS-CoV-2 and its in vitro blockage was associated with a substantial reduction of viral infectivity [14, 15]. These evidences may support the hypothesis that miRNA-3115 and miRNA-7151 expression could represent a protective factor against worse COVID-19 through the inhibition of the pathways mentioned above.

Besides miRNAs effects, in our cohort, vitamin D deficiency was previously demonstrated to predict the occurrence of severe outcomes prospectively and independently [6]. Several pathophysiological hypotheses were proposed to explain the negative influences of vitamin D deficiency in COVID-19 [1, 16–19], however, no data are currently available regarding vitamin D and miRNAs relationship in this clinical setting. Despite the non-statistically significant differences in either rates of hypovitaminosis D or 25(OH) vitamin D levels among patients with and without miRNAs expression, possibly also due to the limited number of study subjects, we found an association between vitamin D deficiency and lack of miRNA-7151 expression in COVID-19 patients with worse outcomes which may support the hypothesis that the co-existence of these two conditions may have a strong negative prognostic role. It remains to be investigated in larger cohorts if lack of vitamin D and miRNA-7151 can act synergistically and independently, or with a cause-effect relationship or even if supplementation of vitamin D may have more effective protective role in those with vitamin D deficiency and lack of protective miRNA expression [20].

First limitation of our study is the low number of patients included. This is mainly related to the stringent inclusion and exclusion criteria adopted. Another important limitation is the lack of evaluation of potential pathophysiological mechanisms underlying the associations between miRNAs expression, vitamin D deficiency, and COVID-19 severity.

However, besides these limitations, this is the first study that specifically evaluated vitamin D and miRNAs relationships in influencing COVID-19 outcome. Moreover, we firstly reported on the expression of two novel miRNAs as potentially useful protective biomarkers of COVID-19 severity and the prospective design used in the study allowed us to hypothesize a combined predictive role of low vitamin D levels and miRNA expression.

In conclusion, the expression of two novel miRNAs was reported for the first-time to be associated with a less severe COVID-19 form and to prospectively predict the occurrence of disease outcome. Moreover, the association observed between vitamin D levels and miRNA expression may potentially expand our knowledge about vitamin D role in acute COVID-19, but further investigations should be also performed in Long COVID scenario and in response to anti-COVID-19 vaccination, settings in which vitamin D levels were recently proposed to have a potential role [21–23].
