# Correction: Novel protective circulating miRNA are associated with preserved vitamin D levels in patients with mild COVID-19 presentation at hospital admission not progressing into severe disease

**DOI:** 10.1007/s12020-024-03939-5

**Published:** 2024-06-27

**Authors:** Luigi di Filippo, Umberto Terenzi, Giovanni Di Ienno, Silvia Trasciatti, Silvano Bonaretti, Andrea Giustina

**Affiliations:** 1 Institute of Endocrine and Metabolic Sciences, San Raffaele Vita Salute University and IRCCS San Raffaele Hospital, Milan, Italy; 2 Galileo Research Srl, Pisa, Italy

Correction to: *Endocrine* 10.1007/s12020-024-03900-6

In this article, the author’s name Andrea Giustina was incorrectly written as Giustina Andrea.

The original article has been corrected.

